# SimSpliceEvol: alternative splicing-aware simulation of biological sequence evolution

**DOI:** 10.1186/s12859-019-3207-5

**Published:** 2019-12-17

**Authors:** Esaie Kuitche, Safa Jammali, Aïda Ouangraoua

**Affiliations:** 1 Department of Computer Science, University of Sherbrooke, 2500 Boulevard de l’Université, Quebec, J1K2R1 Canada; 2 Department of Biochemistry, University of Sherbrooke, 3001 12e avenue Nord, Quebec, J1H5N4 Canada

**Keywords:** Simulation, Exon-intron structure, Alternative splicing, Evolution

## Abstract

**Background:**

It is now well established that eukaryotic coding genes have the ability to produce more than one type of transcript thanks to the mechanisms of alternative splicing and alternative transcription. Because of the lack of gold standard real data on alternative splicing, simulated data constitute a good option for evaluating the accuracy and the efficiency of methods developed for splice-aware sequence analysis. However, existing sequence evolution simulation methods do not model alternative splicing, and so they can not be used to test spliced sequence analysis methods.

**Results:**

We propose a new method called SimSpliceEvol for simulating the evolution of sets of alternative transcripts along the branches of an input gene tree. In addition to traditional sequence evolution events, the simulation also includes gene exon-intron structure evolution events and alternative splicing events that modify the sets of transcripts produced from genes. SimSpliceEvol was implemented in Python. The source code is freely available at https://github.com/UdeS-CoBIUS/SimSpliceEvol.

**Conclusions:**

Data generated using SimSpliceEvol are useful for testing spliced RNA sequence analysis methods such as methods for spliced alignment of cDNA and genomic sequences, multiple cDNA alignment, orthologous exons identification, splicing orthology inference, transcript phylogeny inference, which requires to know the real evolutionary relationships between the sequences.

## Background

Alternative splicing is used by eukaryotic coding genes to diversify their transcript production [1]. Splicing [2] is a mechanism by which a primary transcript from a gene undergoes cutout and ligation steps that lead to the elimination of some segments from the transcript to result in a final mature transcript. The segments conserved in the mature transcript are called exons and those that are eliminated by splicing are called introns. Thus, the exon-intron structure of a eukaryotic gene refers to the succession of alternating exon and intron segments that compose the gene sequence. Alternative splicing allows genes to produce several isoforms of transcripts composed of different combinations of exons [2].

The gain and loss of introns and exons in gene structures along the evolution have been studied in various lineages of eukaryots. Intron loss and gain by unknown mechanisms were detected in several lineages [3–5]. Exon loss and gain were also observed in various lineages by mechanisms including genomic deletion, insertion or duplication, and mutational disabling or acquisition of splice sites [6–8]. It was estimated that approximately 95% of multiexonic human genes give rise to alternative splicing [9]. Evolutionary comparisons of alternative exons and transcripts have shown a significant enrichment for evolutionary conservation, and ancient origins of alternative exons [10, 11]. The functional consequences of changes in exon-intron structure and splicing patterns have been widely documented. It was shown that the majority of alternative splicing events display tissue-dependent variation, and give rise to protein functional changes [12–14].

Several methods have been developed for the analysis of spliced transcript sequences. These include methods for the computation of spliced alignment between spliced transcript and unspliced genomic sequences [15–17], the computation of multiple alignment of spliced cDNA sequences [18–20], the identification of orthologous exons in a set of transcripts, the inference of splicing orthology relations between transcripts [21, 22], the reconstruction of alternative transcript phylogenies [23–25], the clustering of proteins, transcripts and genes sequences [26–29], to mention only those. However, the lack of real gold standard data for which the true evolutionary relationships between data are known is an obstacle to the evaluation of the performance of methods for spliced transcript sequences analysis. Thus, sequence evolution simulation constitutes a promising avenue for the generation of simulated benchmark data to test these methods.

A multitude of tools have been developed for the simulation of the evolution of biological sequences [30–38]. Most of these methods take as main input a guide tree, generate an ancestral sequence at the root of the guide tree, and make this sequence evolve iteratively along the branches of the tree. The simulated evolution events include sequence insertion and deletion (indel) events and substitution events. At the end of the simulated evolution, each leaf of the input guide tree is associated to one sequence for which the full evolutionary history is known. Some sequence evolution simulation tools like PhyloSim [30], indel-Seq-Gen [32] and INDELible [38] simulate the exon-intron structure of genes by defining partitions that evolve under different models and parameters. However the initial exon-intron structure generated at the root the tree can not be modified along the evolution. Some other tools are dedicated to the simulation of raw amino acid sequences [31, 36] or nucleotide sequences [35] evolution without the underlying exon-intron structure of genes. All simulation tools return as result the true alignment of the simulated sequences, which is useful as benchmark data to test sequence analysis methods. However, no existing tool simulates both changes in the exon-intron structure of genes and the alternative splicing mechanisms that drive the evolution of sets of transcripts produced from genes.

In this paper, we present a new simulation tool, called SimSpliceEvol for gene and alternative transcript sequence evolution. SimSpliceEvol simulates events acting on the evolution of the exon-intron structure of genes and alternative splicing events acting on the sets of transcripts produced from genes, in addition to traditional sequence substitution and indel events. SimSpliceEvol takes as input a guide gene tree with branch lengths representing the number of substitutions per site on branches, and generates a set of gene sequences representing a gene family with the exon-intron structures and the sets of cDNA sequences associated to alternative transcripts of the genes. For all simulated gene and cDNA sequences, the true multiple sequence alignment and the orthology relationships between exons and between transcripts are also given as output. Data produced by SimSpliceEvol can be used to evaluate models and methods for spliced sequence analysis. For instance, in [39], we used it to generate simulated data for the comparison of spliced alignment methods. To the best of our knowledge, SimSpliceEvol is the first sequence evolution simulation tool that integrates the simulation of both the evolution of gene exon-intron structure and alternative splicing events.

The paper is organized as follows. The next section is dedicated to the description of the simulation model of SimSpliceEvol. In the “Results” section, a comparison of SimSpliceEvol with existing sequence evolution simulation tools is provided. The usefulness of SimSpliceEvol is illustrated by the use of simulated data to compare the performance of methods for multiple cDNA/protein sequence alignment and methods for cDNA/protein clustering.

## Materials and methods

SimSpliceEvol takes as input a guide gene tree with branch lengths, and generates the genes and cDNA sequences at the leaves of the tree with the true evolutionary history of the gene family. The method starts by simulating an ancestral gene sequence with exon-intron structure and a set of alternative cDNA sequences at the root of the tree. Next, two models of evolution are applied jointly to simulate the evolution from the ancestral gene along branches of the guide tree. The first model makes evolve the exon-intron structure of the genes and the resulting sets of alternative cDNA sequences, and the second model is a codon / nucleotide sequence evolution model by substitution and indel events for the exon and intron sequences.

In this section, we first describe how the ancestral gene sequence at the root of the tree is simulated. Next, we describe the simulation models used on the one hand for the evolution of the exon-intron structure of genes and the sets of alternative cDNA, and on the other hand for the evolution of the exon and intron sequences.

### Root ancestral gene simulation

**Fig. 1:**
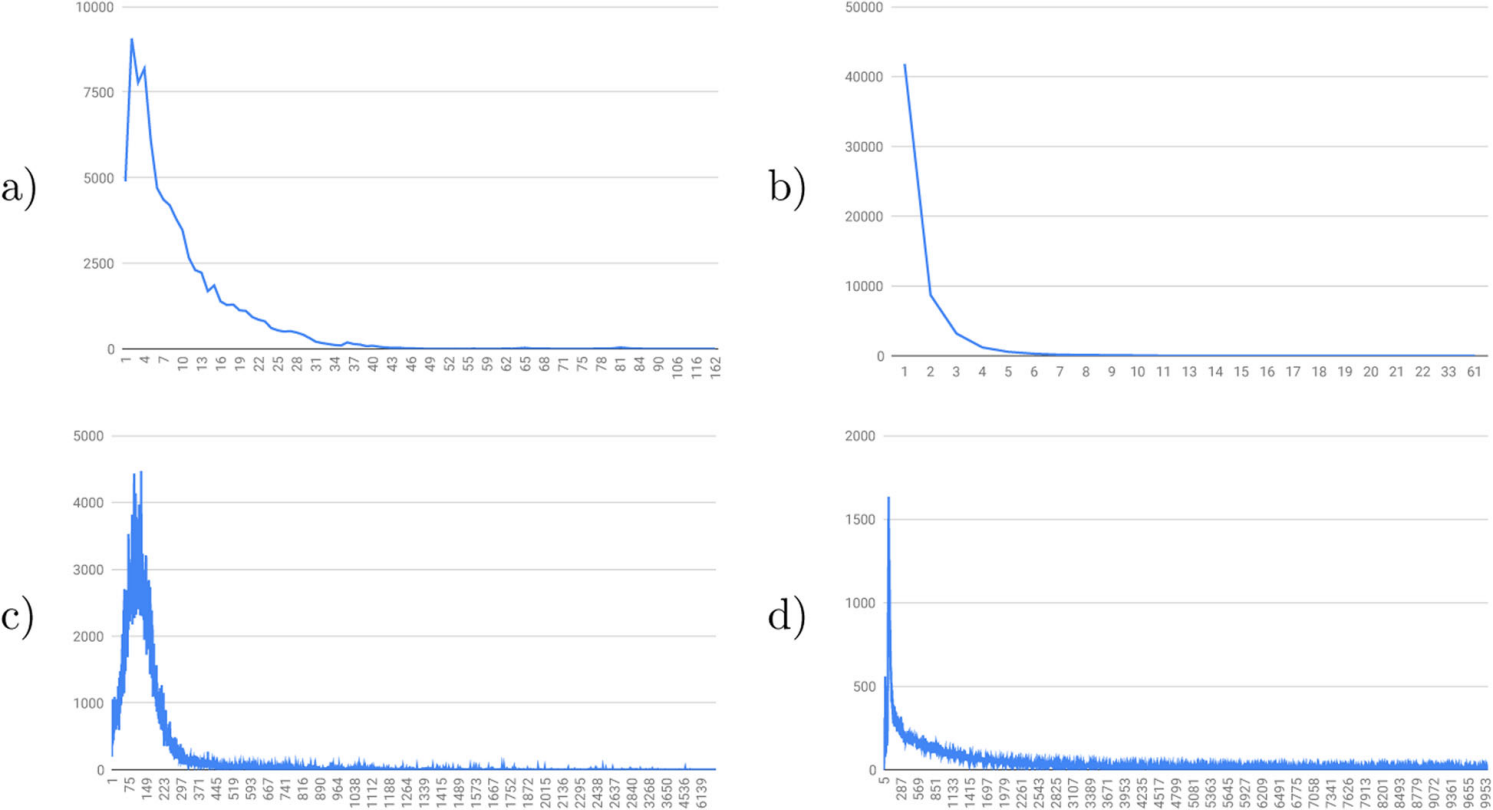
Probability distributions of the composition of gene sequences derived from Ensembl Compara 96 coding genes. **a** Number of exons per cDNA transcript (mean: 9.62; std:8.54). **b** Number of cDNA transcripts per gene (mean: 1.45; std:1.08). **c** Exon length (mean: 170.36; std:258.86). **d** Intron length (mean: 3730.30; std:20126.39)

In order to generate realistic data, we collected the exon-intron structure, transcript and sequence information from all coding genes from the Ensembl Compara 96 database [40]. These dataset is composed of coding genes and cDNA sequences of 189 eukaryotic species. *Oryzias_latipes* has the highest fraction of genes 1.30%, with a median value of 0.55% for all species. *Homos_sapiens* has the highest fraction of transcripts 2%, with a median value of 0.53% for all species. From this data, we derived the probability distributions of the number of exons per cDNA transcript, the number of alternative cDNA transcripts per gene, the length of exon segments, the length of intron segments, and the pair of dinucleotides at the extremities of an intron segment called splice sites. Figure 1 presents the probability distributions obtained.

The nucleotide sequences of translated exons and introns from the Ensembl coding gene dataset were used to build Markov chains to simulate exon and intron sequences. For exon segments, since the interest is to simulate cDNA sequences composed of translated exons, we derived from the data the probabilities for each nucleotide to be in the first position of a codon, to be in the second position given the nucleotide at the first position, and to be in the third position given the dinucleotide at the two first positions of a codon. The derived probabilities used as transition probabilities for the Markov chains are shown in Table 1.

**Table 1:** Markov chain transition probabilities: probability for a nucleotide A, C, T or G to be: a) in the first position *x*_1_ of a codon, b) in the second position *x*_2_ of a codon given the nucleotide at *x*_1_, and c) in the third position *x*_3_ of a codon given the dinucleotide at *x*_1_*x*_2_

a)					b)					c)				
*x*_1_	A	C	T	G	*x*_2_	A	C	T	G	*x*_3_	A	C	T	G
Start					*x*_1_					*x*_1_*x*_2_				
*∅*	0.24	0.31	0.19	0.25	A	0.21	0.28	0.21	0.30	AA	0.16	0.35	0.13	0.36
					C	0.27	0.38	0.21	0.14	AC	0.22	0.49	0.14	0.15
					T	0.12	0.35	0.20	0.33	AT	0.02	0.41	0.14	0.43
					G	0.29	0.30	0.19	0.22	AG	0.12	0.55	0.20	0.13
										CA	0.09	0.22	0.12	0.57
										CC	0.26	0.35	0.28	0.11
										CT	0.07	0.26	0.12	0.55
										CG	0.17	0.37	0.20	0.26
										TA	0.01	0.64	0.34	0.01
										TC	0.20	0.48	0.20	0.12
										TT	0.10	0.48	0.22	0.20
										TG	0.05	0.41	0.30	0.24
										GA	0.21	0.25	0.18	0.36
										GC	0.15	0.55	0.18	0.12
										GT	0.08	0.30	0.16	0.46
										GG	0.19	0.39	0.13	0.29

For intron segments, since the sequences are non-coding, the learning based on codons is not relevant. We simply computed the probabilities for each nucleotide to appear in an intron sequence, and used them to build a zero-order Markov chain. The probabilities of nucleotides are shown in Table 2.

**Table 2:** Probability for a nucleotide A, C, T or G to appear in an intron

A	C	T	G
0.29	0.20	0.30	0.21

**Fig. 2:**
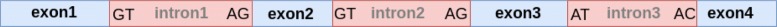
Illustration of a gene structure. A gene structure defined by the succession of alternating exon segments and intron segments with splice sites at their extremities

Based on the probability distributions derived for the structure and the sequence composition of genes, the gene exon-intron structure and nucleotide sequence with a set of coding transcripts are generated for the root of the tree. The exon-intron structure of a gene is defined by the succession of alternating exon and intron segments that compose the gene sequence. Intron segments have specific dinucleotides at their extremities called splice sites that are recognized by the splicing machinery. In 98% of the cases, these dinucleotides are the canonical splice sites GT-AG and in 1% of the cases the non-canonical splice sites AC-AT. (See Fig. 2 for an illustration) [41, 42].

From the probability distributions (Fig. 1), the method first defines the number of exons of the gene. The maximum number *m* of exons per cDNA is sampled, and the number of exons of the gene is defined as *k*_*nbexons*_×*m* where *k*_*nbexons*_≥1 is a user-defined constant. Next, the length of each exon and each intron and the splice sites associated to each intron are sampled. Once the exon-intron structure is generated, the nucleotide sequence for each exon and each intron is generated using the Markov chains built from the nucleotide probabilities (Tables 1 and 2). An exon sequence is built as a chain of independent codons generated one by one once until the length of the exon is reached. Each codon is built progressively using three Markov chains. The zero-order Markov chain is used to generate the first nucleotide *x*_1_ of the codon, and then the first-order Markov chain is used to generate the second nucleotide *x*_2_, and finally the second-order Markov chains is used to generate the third nucleotide *x*_3_. Note that the model imposes that the length of an exon is a multiple of 3. An intron sequence is built as a chain of independent nucleotides flanked by splice sites chosen from the empirical splice site distribution of 98% for GT-AG, 1% for GC-AG, and 1% for all other types of splice sites. The nucleotides of an intron are generated one by one until the length of the intron is reached.

**Fig. 3:**
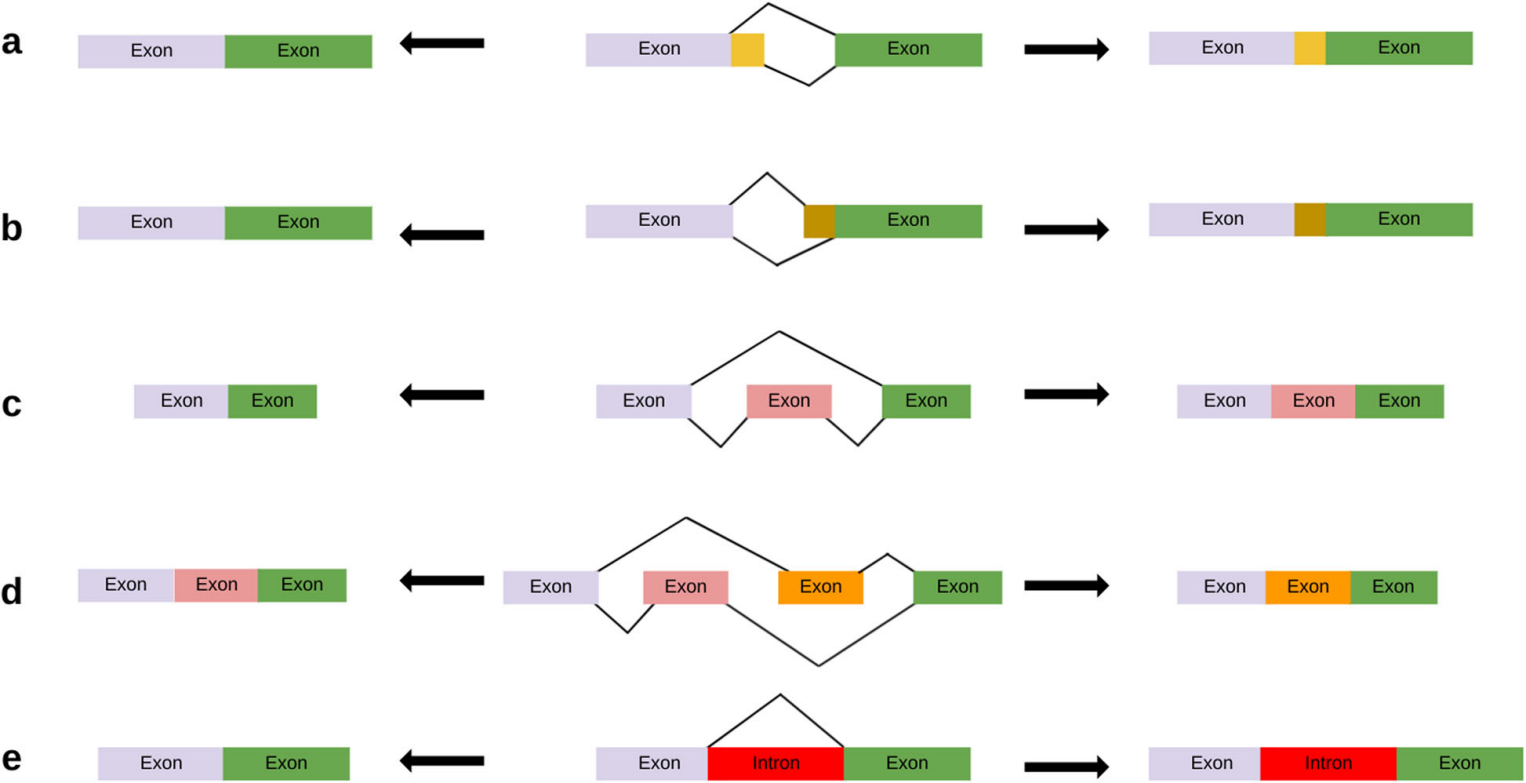
Five main types of alternative splicing events. **a**, **b** Alternative 3’ and 5’ splice-site selections. **c** Exon skipping. **d** Mutually exclusive exons. **e** Intron retention

**Fig. 4:**
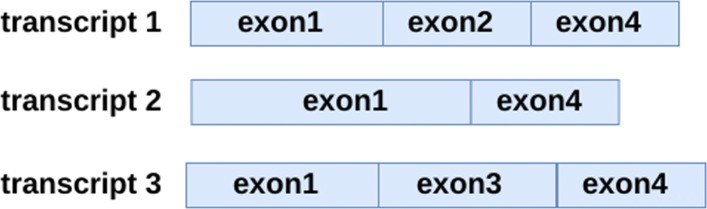
Example of 3 transcripts produced from the gene on Fig. 2 by alternative splicing. The difference between Transcripts 1 and 2 is an alternative 3’ splice-site selection for Exon 1 and an exon skipping for Exon 2. The difference between Transcripts 1 and 3 is an event of mutually exclusive exons for Exons 2 and 3. The difference between Transcripts 2 and 3 is an alternative 3’ splice-site selection for Exon 1 and an exon skipping for Exon 3

Alternative splicing allows genes to produce several isoforms of transcripts composed of different combinations of exons [2]. There exist five main types of elementary alternative splicing events that explain the difference between two transcripts produced from a gene. Alternative 3’ or 5’ splice-site selections occur when two distinct splice sites are used in the intron at the 5’ or 3’ extremity of an exon. Exon skipping is the alternative inclusion or skipping of an exon in the transcripts. Mutually exclusive exons occur when alternatively one of two successive exons is included but not both. Intron retention is the alternative inclusion or splicing of an intron in the transcripts. Note that two transcripts of a gene may differ by a combination of several alternative splicing events. See Fig. 3 for an illustration of the five main types of alternative splicing events and Fig. 4 for an illustration of the production of several isoforms of transcripts from a gene by alternative splicing.

The set of alternative transcripts produced at the root of the guide tree is generated first by randomly selecting a subset of the transcripts from the set of all possible isoforms that have a number of exons less or equal to the maximum number *m* of exons per cDNA. Next, the remaining transcripts are generated by applying alternative splicing events on the transcripts selected randomly.

Six user-defined parameters are used to define the proportion of transcripts generated by random selection or by alternative splicing. They are the relative frequencies of transcript generation by random selection *tc*_*rs*_, alternative 5’ splice-site selection *tc*_*a*5_, alternative 3’ splice-site selection *tc*_*a*3_, exon skipping *tc*_*es*_, mutually exclusive exons *tc*_*me*_, and intron retention *tc*_*ir*_. In the case where *tc*_*rs*_=0, a first transcript is generated by random selection and the other transcripts are generated by alternative splicing. In the case of alternative 3’ and 5’ splice-site selections, the dinucleotide at the new splice site is modified in order to correspond to a known type of splice site.

Once the root gene and its set of alternative cDNA are simulated, the next step is to simulate their evolution along the branches of the guide tree.

### Evolution simulation

SimSpliceEvol combines two models of evolution that are applied conjointly. The first model is the structure evolution model that acts on the evolution the exon-intron structure and the resulting set of transcripts of the gene. The second model is the sequence evolution model that acts on the evolution of the gene sequence. The length of a branch in the input guide tree represents the expected number of substitution events per codon in coding sequences on the branch. To define the expected numbers of any other type of evolutionary events acting on the exon-intron structure, the set of transcripts or the sequence along a branch, we use a linear model, which assumes that all rates are linearly related to the substitution rate.

In the following, we present the two models in two different sections for the sake of clarity, even if there are applied conjointly along branches of the guide tree.

#### Structure evolution model

The structure evolution model used in SimSpliceEvol is an extension of the Christinat-Moret model of transcript evolution introduced in [24]. The model is divided in two levels of evolution, one acting at the gene level on the gene exon-intron structure and the other at the transcript level on the sets of transcripts produced from a gene. We describe below the evolutionary events included in the model at each level.

##### Evolutionary events acting on the exon-intron structure of genes.

The evolution of the gene exon-intron structure is driven by three elementary events that are the loss, gain and duplication of an exon. The loss of an exon occurs when an exon is removed from the gene exon-intron structure and does not belong anymore to any transcript of the gene. The loss of an exon is implemented as a deletion of the exon segment from the gene sequence. The gain of an exon is the appearance of a new exon segment inside an existing intron. It is implemented by generating a new exon segment and inserting it inside an existing intron segment. The duplication of an exon consists in a tandem duplication of an exon segment and the insertion of an intron segment between the two copies. The application of multiple successive events along a branch of the guide tree leads to a modification of the exon-intron structure of the gene.

Given a branch of the guide tree with length *codon*_*subst*_*rate* representing the expected number of substitution events per codon on the branch, the expected number of exon-intron structure change (EIC) events per exon on the branch is calculated as *k*_*eic*_×*codon*_*subst*_*rate* where *k*_*eic*_ is a user-defined constant. Three additional user-defined parameters are used to define the proportion of EIC events acting on the evolution of the exon-intron structure of genes. They are the relative frequencies of exon-intron structure change by exon loss *eic*_*el*_, exon gain *eic*_*eg*_, and exon duplication *eic*_*ed*_, such that the sum of these three relative frequencies equals 1.0. Thus, for example, the overall expected number of exon loss events on a gene having *n* exons is calculated as *n*×*eic*_*el*_×*k*_*eic*_×*codon*_*subst*_*rate* for a branch of length *codon*_*subst*_*rate*.

##### Evolutionary events acting on the set of transcripts of genes.

The evolution of the sets of transcripts produced from genes are driven by the evolution of the gene exon-intron structure, and also by events that take place at the transcript level. Two elementary events can affect the set of transcripts produced from a gene at the transcript level: the loss and the creation of a transcript.

The loss of a transcript occurs a gene stops to produce a given type of transcript due to mutations or a new regulation of the gene expression. The simulation of a transcript loss event consists in stopping the generation of a transcript starting from the node where it is lost. Note that the model makes the assumption that the loss of an exon at the gene level implies a loss of all the transcripts that contain this exon.

The creation of a transcript occurs when a gene starts producing a new type of transcript, i.e., a new combination of exons from its exon-intron structure. Alternative splicing determines which alternative exons are present or absent in each transcript of a gene. The simulation of a transcript creation event in the model consists in generating a new transcript either randomly from the set of all possible isoforms, or by applying an alternative splicing event on an existing transcript (See Fig. 3 for an illustration of the five main types of alternative splicing events).

Given a branch of the guide tree with length *codon*_*subst*_*rate*, the expected number of transcript change (TC) events per transcript on the branch is calculated as *k*_*tc*_×*codon*_*subst*_*rate* where *k*_*tc*_ is a user-defined constant. The six user-defined parameters *tc*_*rs*_,*tc*_*a*5_,*tc*_*a*3_,*tc*_*es*_,*tc*_*me*_,*tc*_*ir*_ used for the generation of transcripts at the root of the guide tree are also used as the relative frequencies of TC events by random selection *tc*_*rs*_, alternative 5’ *tc*_*a*5_ and 3’ *tc*_*a*3_ splice-site selection, exon skipping *tc*_*es*_, mutually exclusive exons *tc*_*me*_, and intron retention *tc*_*ir*_. An additional user-defined parameter, the relative frequency of TC events by transcript loss *tc*_*tl*_ is used to define the relative proportion of TC events by transcript loss, such that the sum of these seven relative frequencies equals 1.0. So, for example, the overall expected number of transcript loss on a gene having *n* transcripts is calculated as *n*×*tc*_*tl*_×*k*_*tc*_×*codon*_*subst*_*rate* for a branch of length *codon*_*subst*_*rate*.

The set of transcripts produced from the exon-intron structure of a gene results in one of the three following states for each exon: absent, alternative, or constitutive. An exon is absent if it does not belong to any transcript of the gene. An exon is alternative if it may be absent or present in transcripts produced from the gene. A constitutive exon is present in all transcripts produced from the gene. Note that along a branch of the guide tree, an exon can transit from any status to another. SimSpliceEvol does not explicitly integrate the simulation of exon status changes. These changes are induced by the comparison of the sets of transcripts generated at the two extremities of a branch.

**Fig. 5:**
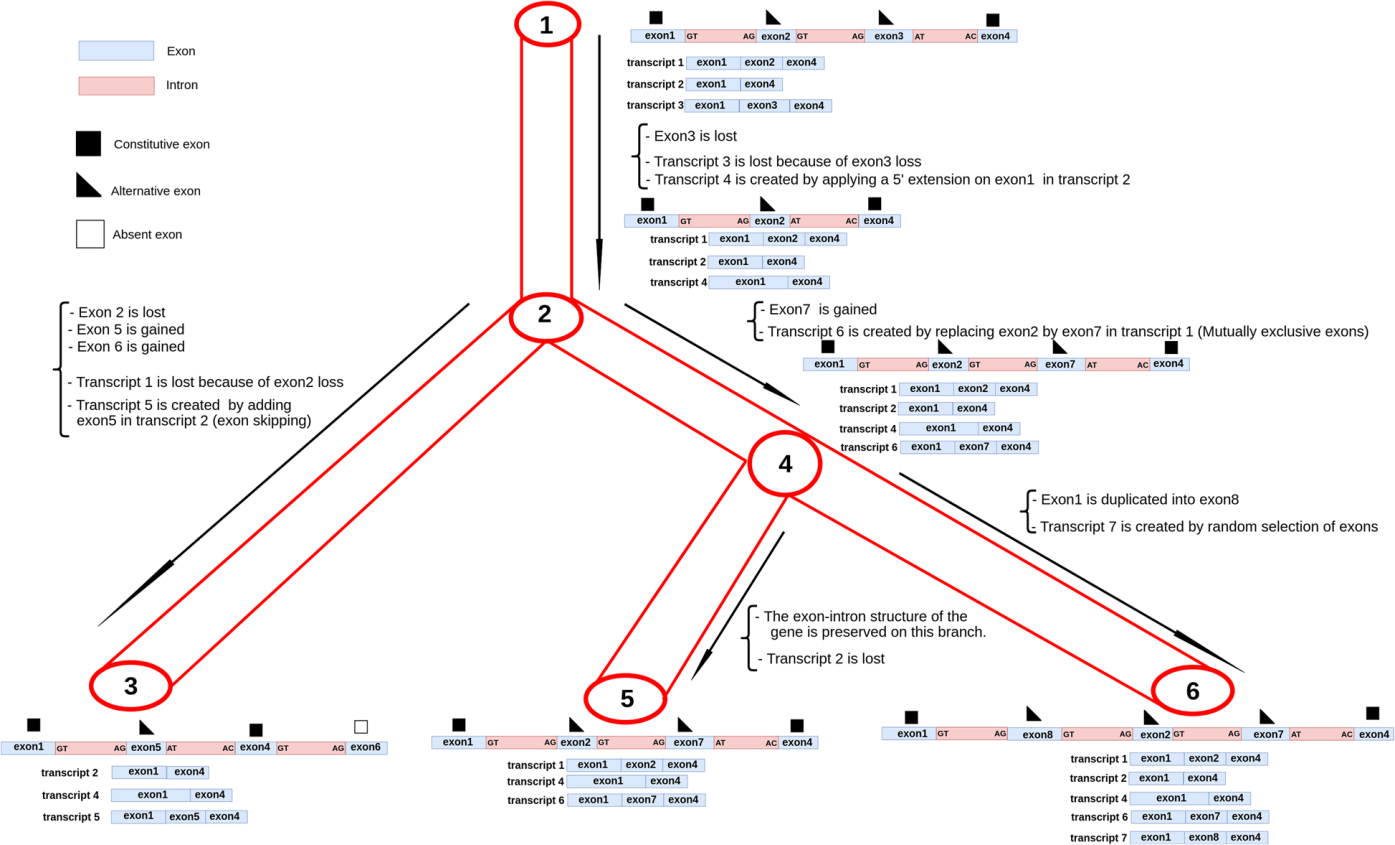
Illustration of SimSpliceEvol model of evolution. Scenario of evolution of the exon-intron structure and the set of transcripts in a gene tree (depicted in red)

Figure 5 illustrates an example of simulation of the evolution of a gene exon-intron structure with the resulting transcripts along branches of a guide tree that has three leaves.

#### Sequence evolution model

In addition to the evolution of the exon-intron structure and the set of transcripts of genes, the sequence of genes also evolve through insertion and deletion (indel) events and substitution events. A multitude of methods have been developed for the simulation of coding and non-coding sequence evolution. For SimSpliceEvol, we did not develop a new sequence evolution simulation model. We used the same sequence evolution simulation models as indel-Seq-Gen [32] for coding exon and non-coding intron sequences.

##### Exon sequence evolution model.

The model includes codon substitution and indel processes. For each exon segment, codon substitution events along a branch of the guide tree are simulated based on the branch length *codon*_*subst*_*rate* that represent the expected number of substitution events per codon on the branch. Each substitution event is generated based on an empirical codon substitution matrix [43] that gives the probability of transition between any two types of codon.

The indels are also simulated based on the branch length *codon*_*subst*_*rate*. The expected number of indel events per codon on the branch is calculated as *k*_*indel*_×*codon*_*subst*_*rate* where *k*_*indel*_ is a user-defined constant. The length of each indel event is drawn from an empirically derived distribution of indel lengths [44]. The codon sequence of an inserted segment is generated using the Markov chains described in Table 1. Two user-defined parameters are used to define the proportion of insertion and deletion events. The relative frequencies of insertion and deletion events are denoted by *ci* and *cd*, such that *ci*+*cd*=1.0. Thus, for instance, the overall expected number of codon deletion events on an exon segment composed of *n* codons is calculated as *n*×*cd*×*k*_*indel*_×*codon*_*subst*_*rate* for a branch of length *codon*_*subst*_*rate*.

##### Intron sequence evolution model.

The model is the same as the exon sequence evolution model, except that the substitution and indels processes are simulated at the nucleotide level, and the expected numbers of substitution and indel events per nucleotide on the guide tree branches are multiplied by a user-defined constant *k*_*intron*_. So, for instance, the overall expected number of nucleotide deletion events on an intron segment of length *n* is *k*_*intron*_×*n*×*cd*×*k*_*indel*_×*codon*_*subst*_*rate* for a branch of length *codon*_*subst*_*rate*.

### Implementation

SimSpliceEvol generates a gene sequence with exon-intron structure and a set of alternative transcripts at the root of the guide tree according to the probability distributions in Fig. 1 and Tables 1 and 2. Next, the program recursively generates the mutations along each branch of the tree from root to leaves, such that each descendant node inherits all the mutations generated along the path between the root and the node. On each branch, exon-intron structure mutations (exon loss, exon gain, and exon duplication) are first performed, then transcript mutations (transcript loss and transcript creation) are performed, and finally sequence mutations (substitution and indels) are performed.

For the simulation of exon-intron structure mutations along a branch (*i*,*j*), the method first computes the overall expected number of exon loss events according to the frequency of exon loss and the number of exon at node *i*, and the exons to be deleted are chosen randomly and removed from the gene. Next, the overall expected number of exon gain events is computed according to the frequency of exon gain and the new number of exons, the insertion positions are randomly chosen, and new exon segments are generated and inserted at these positions. Finally, the overall expected number of exon duplication events is computed according to the frequency of exon duplication and the new number of exons, and the exons to be duplicated are chosen randomly and duplicated.

The simulation of transcript set mutations is done by first performing all transcript loss events and then transcripts creation events by random selection or by one the five alternative splicing event types. For each type of event, the overall expected number of events is adjusted to the number of transcripts at the moment of the simulation.

The simulation of the evolution of each exon and intron sequence is performed independently from the other segments. For exon sequences, the codon evolution model is used, and deletions and insertions are performed before substitutions. As for structure evolution mutations, the overall expected number of a type of event is adjusted to the number of codons in the exon at the moment of the simulation. The position of each event is chosen randomly. For intron sequences, the simulation is performed at the nucleotide level following the same steps as for the exon sequences.

The program outputs the gene sequences at leaves of the guide tree, the set of cDNA sequences for each gene with their exon composition and the location of exons in the gene sequence. SimSpliceEvol also outputs all groups of splicing orthologs that are groups of cDNA transcripts descending from the same ancestral transcript without any alternative splicing events in their evolutionary history from the ancestral transcript. For example in Fig. 5, there are six splicing ortholog groups corresponding to Transcripts 1, 2, 4, 5, 6, 7, and the only group with a copy in each gene is the group of Transcript 4. Finally the program keeps track of all the evolutionary events simulated along branches of the tree. This information is used to generate and output the true multiple alignment of all gene and cDNA sequences simulated.

The default values of user-parameters are set as follows, *k*_*nbexons*_=1.5, *k*_*eic*_=*k*_*tc*_=5, *eic*_*el*_=0.4, *eic*_*eg*_=0.5, *eic*_*ed*_=0.1, *tc*_*rs*_=0.05, *tc*_*a*5_=*tc*_*a*3_=*tc*_*me*_=0.1, *tc*_*es*_=0.2, *tc*_*ir*_=0.05, and *tc*_*tl*_=0.4. The default values were chosen to allow an increase of the numbers of exons and transcripts from the root to the leaves of the guide tree, and also based on results from the literature regarding the levels of alternative splicing among eukaryotes [45–47]. For instance, it has been shown that intron retention (*tc*_*ir*_) is the rarest type of alternative splicing, whereas exon skipping (*tc*_*es*_) is the more prevalent [45]. The number of user-parameters is intentionally kept large in order to allow the users to simulate and test various frequencies for the evolution events included in the models.

## Results

### Comparison with existing simulation methods

Table 3 presents a comparison of SimSpliceEvol with existing sequence evolution simulation tools based on criteria used in [32]. The criteria are related to the type of simulated data, simulated evolution and indel treatment. We also consider additional criteria related to exon-intron structure and alternative splicing simulation. Eight simulation methods are compared: ROSE [36], Dawg [35], SIMPROT [31], EvolveAGene3 [37], INDELible [38], indel-Seg-Gen v2.0 (iSGv2.0) [32], PhyloSim [30] and SimSpliceEvol.

**Table 3:** Comparison of 8 sequence simulation methods

	ROSE	Dawg	SIMPROT	EvolveAGene3	iSGv2.0	INDELible	PhyloSim	SimSpliceEvol
Data simulated								
Exon-intron structure					X	X	X	X
Splice sites							X	X
Alternative transcripts								**X**
Coding sequence				X	X	X	X	X
Non-coding sequence	X	X		X	X	X	X	X
Protein sequence	X		X	X^a^	X	X	X	X^a^
Sequence alignment	X	X	X	X	X	X	X	X
Splicing ortholog groups								**X**
Evolution simulated								
Exon-intron structure								**X**
Splice sites							X	
Alternative transcripts								**X**
Coding sequence				X	X	X	X	X
Non-coding sequence	X	X		X	X	X	X	X
Protein sequence	X		X		X	X	X	
Heterogeneous evolution (partition)		X	X		X	X	X	X^b^
lineage-specific motif conservation					X			
Length-specific motif conservation	X				X			
Site-specific motif conservation					X	X	X	
Indel treatment								
Continuous	X	X	X	X				X
Dynamic length adjustment		X			X	X	X	X
Event tracking					X	X	X	X
Probability of ins and del independent	X	X		X	X	X	X	X
Empirical length distribution		X	X	X	X	X	X	X

^a^ The methods can generate amino acid sequences, but the simulation is done only at the nucleotide level. ^b^ The method defines partitions that evolve under two distinct models for exon and intron, but within an exon or an intron segment, the evolution is homogeneous. Features that are specific to SimSpliceEvol are indicated in bold characters

The first set of criteria is related to the data generated for the root and the leaves of the guide tree. Four methods, ISGv2, INDELible, Phylosim, and SimSpliceEvol allow the generation of the exon-intron structure of genes, but only the last two allow the generation of splice sites at the extremity of introns. SimSpliceEvol is the only method that generates alternative transcripts and splicing ortholog groups.

The second set of criteria is related to the evolution models integrated in the methods. Among the four methods that allow the generation of gene exon-intron structure, only SimSpliceEvol allows evolving this structure and the resulting set of alternative transcripts. However, it does not include an evolution model for splice sites, as Phylosim does. The main limitation of SimSpliceEvol is that it does not include models for motif conservation, which is important for the simulation of highly diverged gene family evolution. SimSpliceEvol also only allows heterogeneous evolution between exons and introns, but not within exons, or within introns. For the current first version of the method, we chose to focus on the development of models for the evolution of the exon-intron structure and the set of alternative transcripts. Several models of motif conservation and heterogeneous evolution used in existing methods will be integrated in subsequent versions of SimSpliceEvol (See [32] for a review of existing models for simulation with motif conservation and heterogeneous evolution).

The last set of criteria concerns the models for indel treatment. As Dawg, SimSpliceEvol combines a continuous generation of indel events with dynamic length adjustment. The continuous model consists in calculating first the number of events based on the sequence length and then generating the events iteratively. The dynamic length adjustment consists in recalculating the number of events after each change in the length of the sequence in order to avoid under-estimating or over-estimating the number of events. In SimSpliceEvol, deletions and insertions are performed before substitutions. Each series of events is simulated using the continuous model, but the number of events for each series is computed based on the length of the sequence at the moment of the generation.

### Application

In order to illustrate the usefulness of SimSpliceEvol for testing spliced sequence analysis methods, we used it to generate 3 datasets of gene families using as guide trees, the following 3 species trees obtained from the Ensembl Compara database [40]. ((((bonobo:0.0031, chimpanzee:0.0025):0.0043, human:0.0066):0.0018, gorilla:0.0087):0.0084, orangutan:0.0173);((rabbit:0.1011, (rat:0.0631, mouse:0.0608):0.0522):0.0019, (gorilla:0.0087, human:0.0084):0.0878);(chicken:0.1295, (opossum:0.1165, ((mouse:0.1149, human:0.0962):0.0001, cow:0.1136):0.0144):0.0101);

The first tree is a species tree of primates that was used to generate a dataset of 30 gene families with highly similar genes, called the “Small” dataset. The second species tree of primates and rodents was used to generate a dataset called “Medium” containing 30 gene families with moderately similar genes. And finally, the last species tree of amniotes was used to generate a dataset called “Large” of 30 gene families. The average percent sequence identity (PID) of pairs of sequences within the families of the 3 datasets are 72% for Small (ranging between 70 and 79%), 52% for Medium (ranging between 50 and 54%), and 40% for Large (ranging between 37 and 41%).

The 3 simulated datasets were then used to compare the performance of methods for multiple cDNA/protein sequence alignment and cDNA/protein clustering.

**Comparing the performance of cDNA alignment methods.** For each of the 90 simulated gene families, the set of all cDNA transcript sequences was aligned using the multiple sequence alignment methods MACSE [18], MAFFT [19], and MUSCLE [20]. MACSE is a multiple sequence alignment program that accounts for the underlying codon structure of protein-coding nucleotide sequences. MAFFT is a popular multiple sequence alignment program based on the identification of homologous regions by the fast Fourier transform. MUSCLE is another popular multiple sequence aligner that uses the log-expectation score to speed up its progressive alignment protocol. Note that the set of multiple sequence alignment methods compared here is not exhaustive, as the aim of this experiment is simply to show the usefulness of SimSpliceEvol for the testing of such methods.

**Fig. 6:**
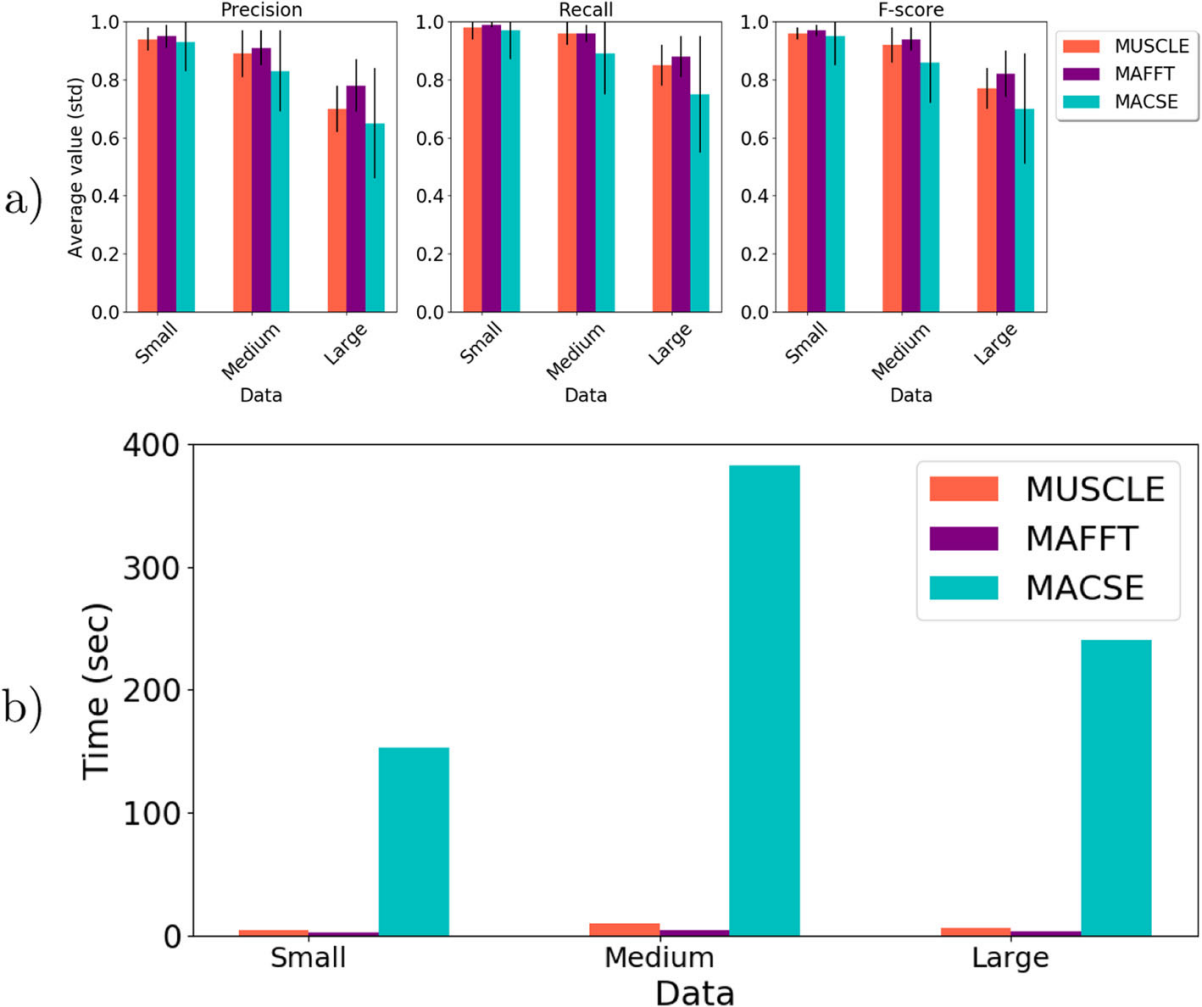
Precision, recall, F-score, and computing time values for multiple sequence alignment methods. Precision, recall, F-score, and computing time values for each dataset (Small, Medium, and Large), and each multiple sequence alignment method (MACSE, MAFFT, and MUSCLE). Precision and recall measures are computed using the true alignment of simulated data as a benchmark. **a** Time (sec), **b** Average value (std)

Using the real alignments of the simulated datasets as a benchmark, the precision, recall, F-score, and computing time values for each method and each gene family were computed. The results compiled by dataset is presented in Fig. 6. The precision measure is the fraction of nucleotide pairs in the estimated alignment that are also in the true alignment. The recall measure is the fraction of nucleotide pairs in the true alignment that are also in the estimated alignment. The F-score is the harmonic mean of precision and recall. We observe that MAFFT is most accurate method with the lowest computing times among the three methods. The accuracy of all methods decreases with the sequence similarity.

**Comparing the performance of cDNA clustering methods.** The proteins generated from the cDNA sequences of the 90 simulated gene families were clustered using the protein sequence clustering methods CLUSS [48], OrthoFinder [27], and OrthoMCL [28]. CLUSS is an alignment-free method for clustering protein families. OrthoFinder is an alignment-based method that infers orthogroups of protein coding genes, by solving the gene length bias in orthogroup inference. OrthoMCL is a popular algorithm for grouping proteins into ortholog groups using pairwise alignment and a Markov Cluster algorithm. It is important to note that none of the three methods was specifically conceived for computing splicing ortholog groups. They were all developed for clustering protein sequences based on their sequences similarities.

**Fig. 7:**
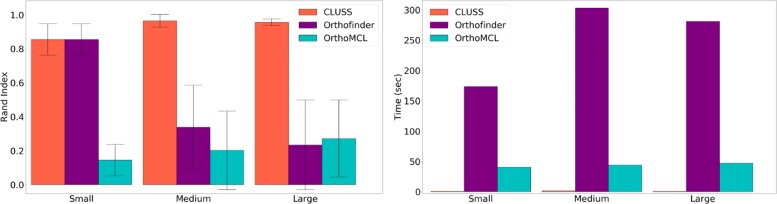
Rand index and computing time values for protein sequence clustering methods. Rand index and computing time values for each dataset (Small, Medium, and Large), and each protein sequence clustering method (CLUSS, OrthoFinder, and OrthoMCL). The Rand index is computed using the true splicing ortholog groups as benchmark

Based on the real splicing ortholog groups of the simulated datasets, the Rand index and the computing time for each method and each gene family were calculated. The results are presented in Fig. 7. The Rand index is the fraction of pairs of protein sequences that have the same relation in the estimated clustering and the real clustering, either in the same cluster or in different clusters. CLUSS obtains the highest Rand index values with the lowest computing times. OrthoFinder obtains the second-best Rand index values with the highest computing times. OrthoMCL has the lowest Rand index values. The performance of OrthoFinder decreases with the similarity of sequences, while the performances of CLUSS and OrthoMCL are robust to changes in sequence similarity. The average real number of clusters in the tree datasets are 3.71 (std: 0.59) for Small, 9.39 (std: 1.58) for Medium, and 10.90 (std: 1.40) for Large. So, the real number of clusters increases with the dissimilarity of sequences. We observed that CLUSS always overestimates the number of clusters with a multiplying factor of 1.90 in average for all datasets. OrthoFinder tends to overestimate or underestimate the number of clusters with average multiplying factors of 1.40, 0.26, 0.17 respectively for the Small, Medium and Large datasets. OrthoMCL always underestimates the number of clusters with average multiplying factors of 0.28, 0.13, 0.15 respectively for the Small, Medium and Large datasets.

## Conclusion

We present a new sequence evolution simulation method called SimSpliceEvol, that simulates the evolution of the exon-intron structure of genes by exon loss, gain and duplication events, and the evolution of the set of alternative transcripts produced from genes by transcript loss events, and transcript creation events through alternative splicing. SimSpliceEvol also simulates traditional indel and substitution evolution events acting at the sequence level. The main added-value of SimSpliceEvol as compared to all existing sequence evolution simulation methods is the evolution of the exon-intron structure and the set of alternative transcripts. The set of user-parameters allows the users to simulate various frequencies for the evolution events included in the models in order to test various hypotheses regarding exon-intron structure and transcript evolution. Through an application, we show the usefulness of SimSpliceEvol for evaluating the performance of spliced sequence analysis methods like multiple sequence alignment methods, and protein sequence clustering methods.

For the first version of the method, we focus on the development of the models for the evolution of the splicing structure of genes and the resulting transcripts. Several additions will be made in subsequent versions of the method to improve the realism of simulated data. First, the method makes two unrealistic assumptions: (1) independence between the codons of an exon sequence, and (2) the length of exons is always a multiple of 3. The first set of additions will relax these assumptions to generate ancestral exon sequences of any length containing known protein motifs. We will also include models for motif conservation, splice sites evolution, and heterogeneous evolution within exon and intron sequences.

The second set of additions concerns the evolution rates on the branches of the guide tree. Currently, the method assumes a linear relation between the evolution rates of all evolution models included in SimSpliceEvol, i.e., sequence evolution, exon-intron structure evolution, and set of transcripts evolution. But, to the best of our knowledge, no empirical study of the relationship between the rates of evolution at the sequence and splicing structure levels has been realized yet. To generalize the model, we will extend the method to allow independent evolution rates at different levels, sequence, transcript, and exon-intron structure.

Finally, the current version of SimSpliceEvol does not include an explicit model for the evolution of exons between absent, alternative, and constitutive states. Future versions will include an explicit model for exon status changes, and the set of exon-intron structure evolution events will be extended to include the loss and gain of an intron.

## Data Availability

Source code available at: https://github.com/UdeS-CoBIUS/SimSpliceEvol. Web server: https://simspliceevol.cobius.usherbrooke.ca.

## Abbreviations

cDNA: Complementary DNA
CDS: Coding DNA sequence
DNA: Deoxyribonucleic acid
